# Supplementary material for: Amylase production from marine sponge Hymeniacidon perlevis; potentials sustainability benefits
Source: PLoS One. 2023 Dec 21;18(12):e0294931. doi: 10.1371/journal.pone.0294931 (PMC10734944; doi:10.1371/journal.pone.0294931)
Supplement: Attachment — Submitted filename: PONE-D-23-14066_comments.pdf [file pone.0294931.s001.pdf]

PLOS ONE

AMYLASE PRODUCTION FROM MARINE SPONGE HYMENIACIDON PERLEVIS; POTENTIALS SUSTAINABILITY BENEFITS

--Manuscript Draft--

**Manuscript Number:**	PONE-D-23-14066
**Article Type:**	Research Article
**Full Title:**	AMYLASE PRODUCTION FROM MARINE SPONGE HYMENIACIDON PERLEVIS; POTENTIALS SUSTAINABILITY BENEFITS
**Short Title:**	AMYLASE PRODUCTION FROM MARINE SPONGE HYMENIACIDON PERLEVIS
**Corresponding Author:**	PRAISE TOCHUKWU Nnaji<br>University of the West of England - Frenchay Campus: University of the West of England<br>BRISTOL, West of England UNITED KINGDOM
**Keywords:**	Amylase; bacteria; sponge; marine; hymeniacidon perlevis; sustainability
**Abstract:**	The marine sponges Hymeniacidon perlevis is a globally distributed and invasive species with extensive filter feeding characteristics. The symbiotic relationship fostered between the sea sponge and the inhabiting microorganism is key in the production of metabolic enzymes which is the focus of this study.<br>Sponge bacterial symbionts were grown on starch agar for 48hrs. Colorimetric analyses of amylase were conducted at 540nm using a spectrophotometric plate reader. The X-Bridge amide 3.5µm, 4.6x150mm column and a mobile phase of 80/20 acetonitrile/water with 0.1% ammonium constituted the liquid chromatography-mass spectrometry (LC-MS) conditions for the analyses. Seven reducing sugars were used to optimise LC-MS to determine the presence of products formed. Some of the bacterial symbionts isolated from H perlevis produced alpha and beta amylases to break down starch. From the statistical mean of product concentrations from the hydrolysis of starch by amylase, isolate 7 had the highest optical density (OD) at 0.43475 while isolate 12 had the lowest OD at 0.141417. From the LC-MS analysis, it was observed that out of the seven sugars, Glucose and maltose constituted > 65% of the products formed from the hydrolysis of starch by the amylases. Isolates 3,6 and 7 produced 6.906 mg/l, 12.309 mg/l, and 5.909 mg/l of glucose, while isolates 3,4,5,6 and 7 produced 203.391 mg/l, 176.238 mg/l, 139.938 mg/l, 39.030 mg/l, and 18.809 mg/l of maltose respectively. Isolate 2 had the highest amount of maltose at a concentration of 267.237 mg/l while isolate 4 had the highest amount of glucose concentration of 53.084 mg/l. Enzymes from marine sponge bacterial offer greater potentials for a green and sustainable production process. Amylase extraction from bacterial symbionts in H perlevis is sustainable and should be encouraged. They can serve as reliable sources of revenue for enzyme industries, and applications in food industries and biotechnological processes.
**Order of Authors:**	PRAISE TOCHUKWU Nnaji
	Emmanuel Adukwu
	H. Ruth Morse
	Rachael U. Chidugu-Ogborigbo
**Additional Information:**	
**Question**	**Response**
**Financial Disclosure**<br><br>Enter a financial disclosure statement that describes the sources of funding for the work included in this submission. Review the submission guidelines for detailed requirements. View published research	R.U. Chidugu-Ogborigbo and P.T Nnaji recieved funding for this work from the University of the West of England, Bristol United Kingdom (https://www.uwe.ac.uk/). P.T. Nnaji and R.U. Chidugu-Ogborigbo designed the study, P.T. Nnaji collected the data collection,P.T. Nnaji, E. Adukwu, H.R. Morse, and R.U. Chidugu-Ogborigbo analysed the data. P.T. Nnaji prepared the manuscript, all authors agreed to publish.

Powered by Editorial Manager® and ProduXion Manager® from Aries Systems Corporation

articles from *PLOS ONE* for specific examples.<br><br>This statement is required for submission and **will appear in the published article** if the submission is accepted. Please make sure it is accurate.<br><br>**Unfunded studies**<br>Enter: *The author(s) received no specific funding for this work.*<br><br>**Funded studies**<br>Enter a statement with the following details:<br>• Initials of the authors who received each award<br>• Grant numbers awarded to each author<br>• The full name of each funder<br>• URL of each funder website<br>• Did the sponsors or funders play any role in the study design, data collection and analysis, decision to publish, or preparation of the manuscript?<br>• **NO** - Include this sentence at the end of your statement: *The funders had no role in study design, data collection and analysis, decision to publish, or preparation of the manuscript.*<br>• **YES** - Specify the role(s) played.<br><br>* typeset	
**Competing Interests**<br><br>Use the instructions below to enter a competing interest statement for this submission. On behalf of all authors, disclose any competing interests that could be perceived to bias this work—acknowledging all financial support and any other relevant financial or non-financial competing interests.<br><br>This statement is required for submission and **will appear in the published article** if the submission is accepted. Please make sure it is accurate and that any funding sources listed in your Funding Information later in the submission form are also declared in your Financial Disclosure statement.	The authors have declared that no competing interests exist.

View published research articles from *PLOS ONE* for specific examples.<br><br>**NO authors have competing interests**<br><br>Enter: *The authors have declared that no competing interests exist.*<br><br>**Authors with competing interests**<br><br>Enter competing interest details beginning with this statement:<br><br>*I have read the journal's policy and the authors of this manuscript have the following competing interests: [insert competing interests here]*<br><br>* typeset	
**Ethics Statement**<br><br>Enter an ethics statement for this submission. This statement is required if the study involved:<br><br>• Human participants<br>• Human specimens or tissue<br>• Vertebrate animals or cephalopods<br>• Vertebrate embryos or tissues<br>• Field research<br><br>Write "N/A" if the submission does not require an ethics statement.<br><br>General guidance is provided below. Consult the submission guidelines for detailed instructions. **Make sure that all information entered here is included in the Methods section of the manuscript.**	N/A

Powered by Editorial Manager® and ProduXion Manager® from Aries Systems Corporation

**Format for specific study types**<br><br>**Human Subject Research (involving human participants and/or tissue)**<br>• Give the name of the institutional review board or ethics committee that approved the study<br>• Include the approval number and/or a statement indicating approval of this research<br>• Indicate the form of consent obtained (written/oral) or the reason that consent was not obtained (e.g. the data were analyzed anonymously)<br><br>**Animal Research (involving vertebrate animals, embryos or tissues)**<br>• Provide the name of the Institutional Animal Care and Use Committee (IACUC) or other relevant ethics board that reviewed the study protocol, and indicate whether they approved this research or granted a formal waiver of ethical approval<br>• Include an approval number if one was obtained<br>• If the study involved *non-human primates*, add *additional details* about animal welfare and steps taken to ameliorate suffering<br>• If anesthesia, euthanasia, or any kind of animal sacrifice is part of the study, include briefly which substances and/or methods were applied<br><br>**Field Research**<br><br>Include the following details if this study involves the collection of plant, animal, or other materials from a natural setting:<br>• Field permit number<br>• Name of the institution or relevant body that granted permission	
**Data Availability**<br><br>Authors are required to make all data underlying the findings described fully available, without restriction, and from the time of publication. PLOS allows rare exceptions to address legal and ethical concerns. See the PLOS Data Policy and FAQ for detailed information.	Yes - all data are fully available without restriction

A Data Availability Statement describing where the data can be found is required at submission. Your answers to this question constitute the Data Availability Statement and **will be published in the article**, if accepted.<br><br>**Important:** Stating 'data available on request from the author' is not sufficient. If your data are only available upon request, select 'No' for the first question and explain your exceptional situation in the text box.<br><br>Do the authors confirm that all data underlying the findings described in their manuscript are fully available without restriction?	
**Describe where the data may be found in full sentences. If you are copying our sample text, replace any instances of XXX with the appropriate details.**<br><br>• If the data are **held or will be held in a public repository**, include URLs, accession numbers or DOIs. If this information will only be available after acceptance, indicate this by ticking the box below. For example: *All XXX files are available from the XXX database (accession number(s) XXX, XXX.).*<br>• If the data are all contained **within the manuscript and/or Supporting Information files**, enter the following: *All relevant data are within the manuscript and its Supporting Information files.*<br>• If neither of these applies but you are able to provide **details of access elsewhere**, with or without limitations, please do so. For example:<br><br>*Data cannot be shared publicly because of [XXX]. Data are available from the XXX Institutional Data Access / Ethics Committee (contact via XXX) for researchers who meet the criteria for access to confidential data.*<br><br>*The data underlying the results presented in the study are available from (include the name of the third party*	This work is part of my PhD research. I intend to use some of the photographs when writing my final thesis. This thesis will be available online at the University website https://www.uwe.ac.uk/

and contact information or URL).<br>• This text is appropriate if the data are owned by a third party and authors do not have permission to share the data.<br><br>* typeset	
Additional data availability information:	

Powered by Editorial Manager® and ProduXion Manager® from Aries Systems Corporation

Manuscript

Click here to access/download;Manuscript;Plosone paper.docx

AMYLASE PRODUCTION FROM MARINE SPONGE *HYMENIACIDON PERLEVIS*; POTENTIALS SUSTAINABILITY BENEFITS

AUTHORS: Praise Tochukwu Nnaji, Emmanuel Adukwu, H. Ruth Morse, and Rachael U. Chidugu-Ogborigbo*

ADDRESS: School of Applied Sciences, College of Health, and Applied Sciences,

University of the West of England Bristol United Kingdom

KEY WORDS: Amylase, bacteria, sponge, marine, *hymeniacidon perlevis*, sustainability

ABSTRACT

The marine sponges *Hymeniacidon perlevis* is a globally distributed and invasive species with extensive filter feeding characteristics. The symbiotic relationship fostered between the sea sponge and the inhabiting microorganism is key in the production of metabolic enzymes which is the focus of this study.

Sponge bacterial symbionts were grown on starch agar for 48hrs. Colorimetric analyses of amylase were conducted at 540nm using a spectrophotometric plate reader. The X-Bridge amide 3.5µm, 4.6x150mm column and a mobile phase of 80/20 acetonitrile/water with 0.1% ammonium constituted the liquid chromatography-mass spectrometry (LC-MS) conditions for the analyses. Seven reducing sugars were used to optimise LC-MS to determine the presence of products formed. Some of the bacterial symbionts isolated from *H perlevis* produced alpha and beta amylases to break down starch. From the statistical mean of product concentrations from the hydrolysis of starch by amylase, isolate 7 had the highest optical density (OD) at 0.43475 while isolate 12 had the lowest OD at 0.141417. From the LC-MS analysis, it was observed that out of the seven sugars, Glucose and maltose constituted > 65% of the products formed from the hydrolysis of starch by the amylases.

Isolates 3,6 and 7 produced 6.906 mg/l, 12.309 mg/l, and 5.909 mg/l of glucose, while isolates 3,4,5,6 and 7 produced 203.391 mg/l, 176.238 mg/l, 139.938 mg/l, 39.030 mg/l, and 18.809 mg/l of maltose respectively. Isolate 2 had the highest amount of maltose at a concentration of 267.237 mg/l while isolate 4 had the highest amount of glucose concentration of 53.084 mg/l. Enzymes from marine sponge bacterial offer greater potentials for a green and sustainable production process. Amylase extraction from bacterial symbionts in *H perlevis* is sustainable and should be encouraged. They can serve as reliable sources of revenue for enzyme industries, and applications in food industries and biotechnological processes.

1.0 INTRODUCTION

Starch is the largest and principal form of which plant polysaccharide is stored in plants. Almost all forms of food plants such as seeds, leaves, tubers, stems, grains etc., have some percentages of starch in them [1]. Starch is constituted of amylose and amylopectin. About 20 to 25% of starch is constituted of amylose while the remaining 75 to 80% of starch constitutes the amylopectin. The linear successive chains of glucose units in starch are linked by the α1, 4-glycosidic bond and the highly branched chains which occurs after 15 to 45 glucose units is linked by the α-1, 6 glycosidic bonds [2, 3].

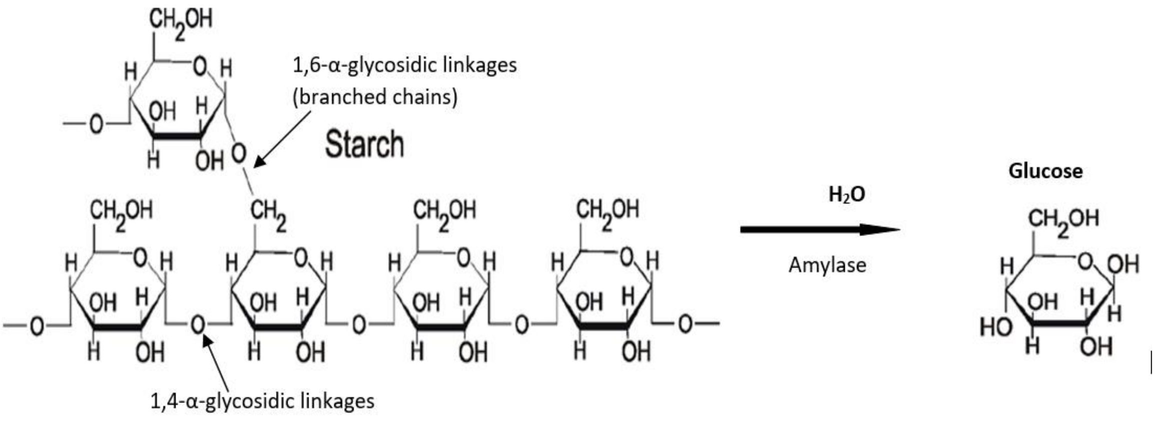

Figure1: Hydrolysis reaction by amylase in the presence of water molecule

Amylases breakdown starch or oligosaccharide molecules to form smaller sugars molecules. They represent a group of enzymes that are extracellularly produced. They are produced by plants, animals, and microorganisms such as bacteria, fungi and actinomyces etc [4]. Amylases in present day are applicable in many production processes of food, pharmaceuticals, detergent, textile industries etc. Amylases have high demand in industries and the world enzyme market. It constitutes approximately about 25 to 30% of the enzyme sold in the global enzyme marke[illegible] Amylases are grouped into four cateories which includes endoamylases, exoamylases, debranching enzymes and transferases [5]. Another classification of amylase is the alpha amylase, beta amylase, isoamylase and glucoamylase [1]. Alpha amylase has the enzyme commission num[illegible] “E.C.3.2.1.1”. It is an endo-amylase that that catalyses the hydrolysis of glycosidic bond (alpha-1,4-glycosidic bonds) in starch to produce glucose and maltose [5]. This enzyme contains calcium as a metalloenzyme and depends on metallic co-factor for its metabolic activities. Alpha-amylases are found naturally in microorganisms, plants, and animals; their main research interest is linked to their ability to hydrolyse starch [2,6,7]. The beta amylase (E.C.3.2.1.2) is produced by microorganisms, and plants like sweet potatoes, soya beans, ray seed and higher plants seeds. The activity of beta amylase involves the hydrolysis of starch to maltose, beta- maltose or beta-limit dextrin. Dextrin units are formed due to inability of beta amylase to hydrolyse branched chains of amylopectin and glycogen [1, 2].

The enzyme industries continue to face increase in the demand for enzymes because of its health, environmental, and economic benefits. Enzymes are also more reliable in biocatalytic activities than inorganic catalyst [8]. Additionally, the processes of enzyme mediated reaction comply with the principles of green and sustainable chemistry more than inorganic catalyst [9-11]. This is because enzyme-based processes offer an environmentally benign catalyst which have selective processes at different levels, and mild or no consequence to the environment at large [11]. They possess high specificity and rate of reaction. Most enzyme producing industries rely on microorganisms for their production of enzyme. Microorganisms

can be cultivated any time and season of the year, they can be manipulated and have fast growth and production process compared to plants and animal as sources of enzymes [12]. Microorganisms are more reliable to offer reduced carbon emission, cleaner and sustainable chemical production processes [13]. However, marine microorganism because of peculiar environmental adaptative traits, have proven to be one of the most reliable sources of enzymes with higher stability to some extreme conditions [14-16].

Sea sponges are good host for marine microorganisms and important members of the benthic and coral reef community. They exist in symbiotic relationship with microorganisms and most benthic aquatic biota. Most sponges possess simple body plan and rely extensively on getting food from their surrounding environments [17,18]. Given their extensive symbiotic relationship with microorganisms, sponges constitute rich sources of key secondary metabolites with unique therapeutic fingerprints [19, 17, 20]. It is reported that in the Caribbean coral reef sponges through complex symbiosis with microorganisms are involved in key roles like primary production, nitrification and largely impact the carbonate framework of the environment [21, 22, 18]. Both the sponge and symbiotic microorganisms produce metabolic enzymes. Sponges are very resilient organisms and are less affected by ocean warming or ocean acidification compared to other benthic organisms, such as corals [19]. They survive and reproduce despite environmental conditions like high salt concentration, low temperatures, high temperatures, high pressure etc [23]. These properties enlist sponges as excellent source for the isolation of bacterial enzyme with biotechnological application in food industries.

2.0 MATERIALS AND METHODS

2.1 Sample collection and storage

With the aid of scalpel blade, sponges were collected randomly during the low tides(~1m) from benthic marine habitat within the coastal rock pools from Tenby Bay (UK). Samples were immediately transported to the laboratory in sea water and immediately preserved until when needed.

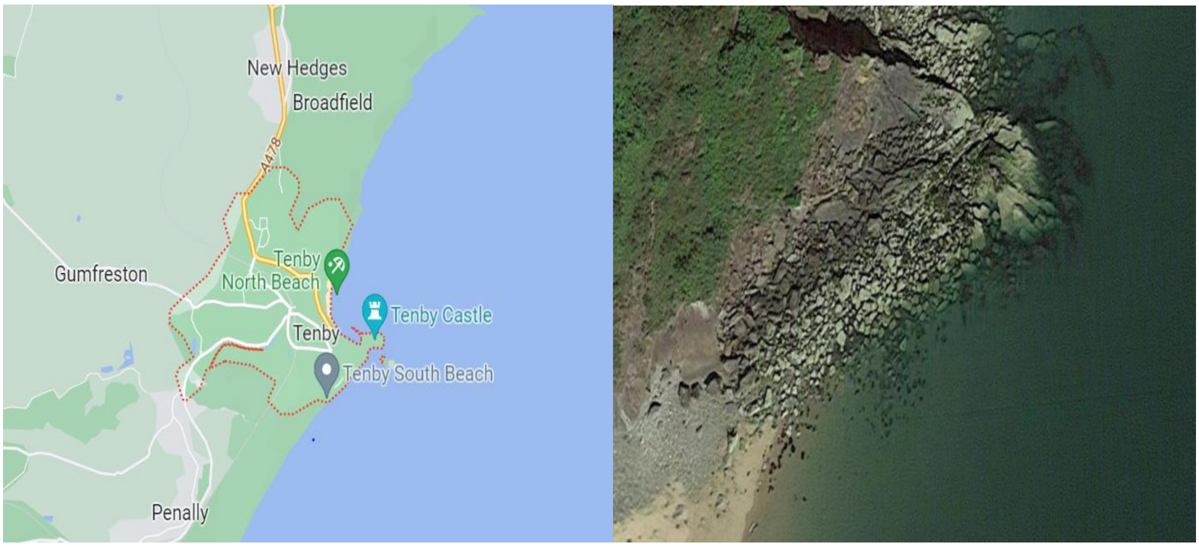

Figure2: Map and Satellite view of sampling site at Tenby Bay Castle beach Pembrokeshire, South Wales (Latitude: 51° 40' 22.04" N; Longitude: -4° 42' 16.09" W).

2.2 BACTERIAL CULTURE AND STAINING

The sponge tissue was blended, and the single cells extracted with the aid of a sterile pipette. Sponge extracts were serially diluted to 10^{-6} following the method described in [24]. There after 500µl aliquot of extract was inoculated into starch agar plate by spread and pour plate method. The plates were incubated at 37^{0}c for 48 hours. The cultures were sub-cultured to obtain a pure culture. Pure culture was gram stained according to standard gram staining procedures.

2.3 STARCH HYDROLYSIS TEST

After 48hours of incubation of bacterial isolates, the hydrolysis of starch was tested by flooding pure culture of bacterial growth with iodine solution. After a few minutes of

observation, zone of clearance around bacterial colony indicated starch hydrolyses by amylase while the lack of zone of clearance with uniform blue-black colouration indicated absence of starch hydrolysis.

2.4 AMYLASE PRODUCTION AND EXTRACTION

Amylase was produced by inoculating pure cultures of positive isolates into nutrient broth and incubated at 37^0c for 24hrs. Afterwards the nutrient broth was lightly vortexed to have a uniform distribution of bacterial growth. 1ml of the broth culture was transferred into amylase extraction medium made up of nutrient broth and soluble starch. This medium was incubated for 24 to 48hours at 37^0c. Afterwards, the culture was centrifuged at 8000g at 4^0c for 20minutes. Thereafter, the supernatant (crude enzyme) was carefully separated from the sediment (bacterial cells) for further analysis.

2.5 COLORIMETRIC ENZYME ASSAY

Amount of product formed from starch hydrolysis was examined using the 3,5-dinitrosalycyclic acid (DNS) method at 540nm according to the method of Fishman, (1984) [25].

2.6 STANDARD CURVE

Standard curve of two sugars (maltose and glucose) were made by preparing standard solutions of maltose and glucose sugars at a concentration of 50, 100, 150, 200 and 250 mg/l.

2.7 LIQUID CHROMATOGRAPHY MASS SPECTROMETRY

The X-Bridge amide 3.5µm, 4.6x150mm column and a mobile phase of 80/20 acetonitrile/water with 0.1% ammonium constituted the liquid chromatography-mass spectrometry (LC-MS) conditions for the analyses. Seven reducing sugars (Maltose,

glucose, sucrose, mannose, fructose, lactose, and galactose) were used to optimise LC-MS to determine the presence of products formed.

2.8 STATISTICS

Data collated from the analyses were inputted into statistical software (SPSS and windows 10 Microsoft excel). They were analysed for normal distribution, thereafter the mean, standard deviation (SD), and standard error of the mean (SEM) were determined. The data generated were used to plot graphs.

3.0 RESULTS

3.1 STARCH HYDROLYSIS TEST

After 48hours of incubation, there were observable growth of mixed colonies with various morphological characteristics. Discrete colonies with uniform morphological characteristics, were sub-cultured into fresh starch agar plates to obtain pure cultures. After flooding the pure cultures with iodine, the hydrolysis of starch was indicated by the zone of clearance around bacterial colony as indicated in figure 3 below.

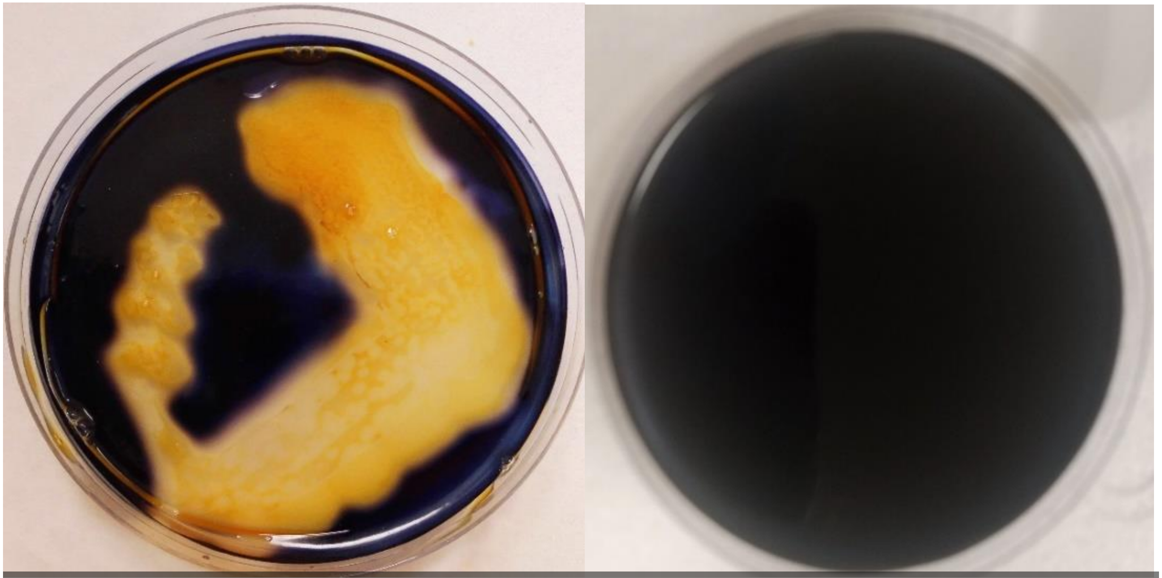

A) Positive amylase test with zone of clearance indicating starch hydrolysis after flooding plate with iodine

B) Negative amylase test with no zone of clearance to indicate starch hydrolysis after flooding plate with iodine

Figure 3: Photograph of positive (plate A) and negative (Plate B) test for starch hydrolysis by flooding growth in plate with iodine.

3.2 GRAM STAIN

All pure culture of isolates from the sponge sample was subjected to gram staining. This technique was adopted to verify bacterial organisms. With the Gram staining, bacteria would retain the purple colour from the primary stain (crystal violet) indicating a gram-positive bacterium as indicated in "A and B". On the contrary, gram-negative bacteria will lose the primary stain upon decolourisation and pick up a counter pink colour from safranin as shown in slide "C".

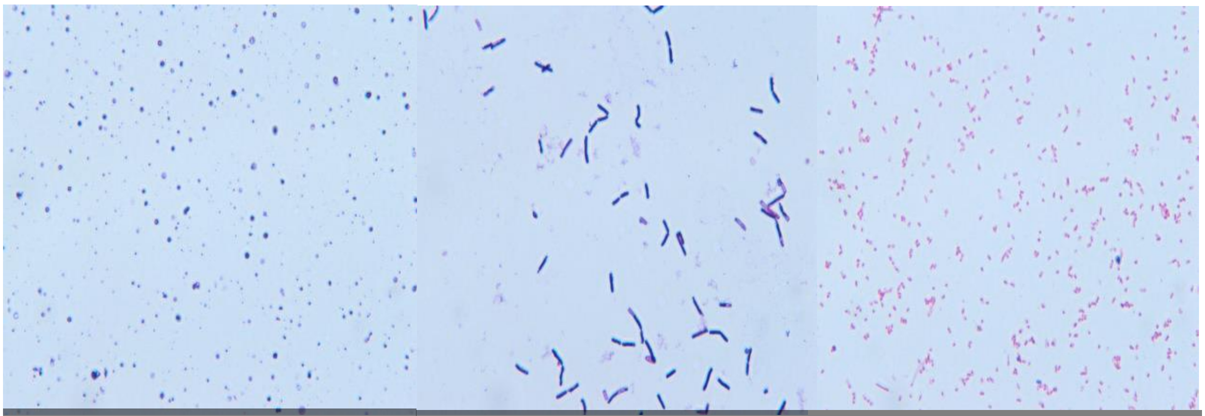

A) Gram positive cocci shaped bacteria

B) Gram-positive Rod-shaped bacteria

C) Slides of gram-negative bacteria

Figure 4: Slides of gram positive and negative bacteria from marine sponge *H perlevis.*

3.3: COLORIMETRIC ENZYME ASSAY

The amount of the reducing sugar produced from the hydrolysis of starch by bacterial amylases was analysed by the 3,5-dinitrosalycyclic acid (DNS) method. The colorimetric assay (Figure 5a) and various absorbances at 540nm from 12 bacterial isolates (figure 5b) are represented below.

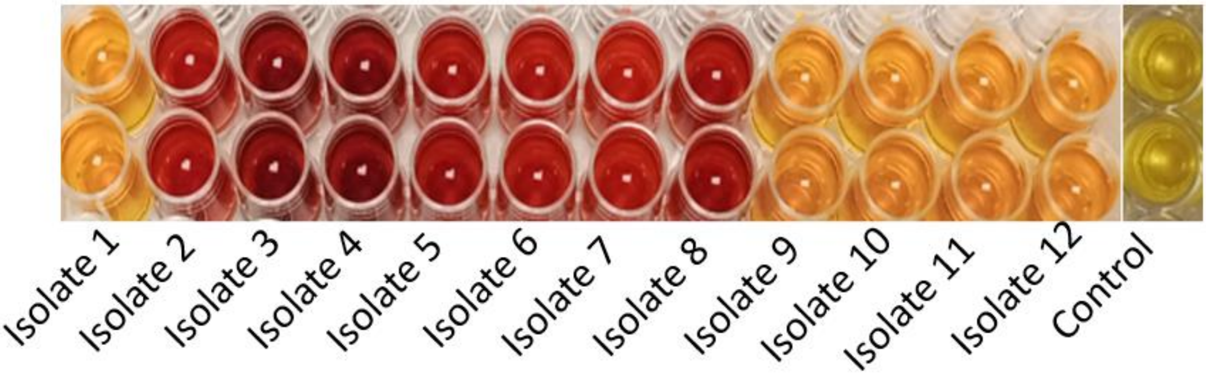

Figure 5a: Reduction reaction indicated by the change of colour from amber yellow to deep brow and a control (DNS and distilled water).

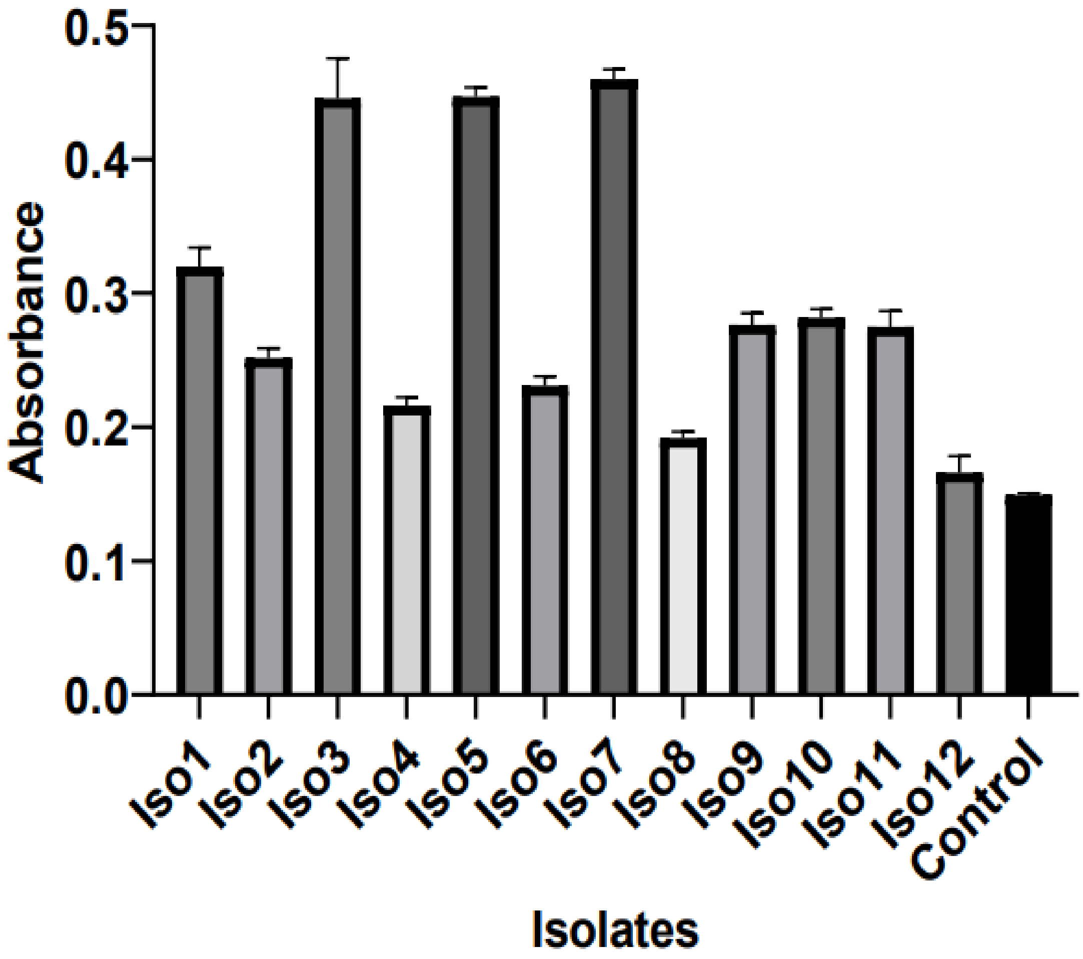

Figure 5b: Graph indicating the quantity of product formed from starch hydrolysis by amylases produced from symbiotic bacteria isolated from *H. perlevis* (n=3).

STANDARD CURVE

To quantify the concentration of glucose and maltose (figure 5 and 6) formed from the hydrolysis of starch by amylase produced from the sponge bacterial isolate, the standard curve below was developed using the X-Bridge amide 3.5µm, 4.6x150mm column for the LC-MS. The graph was plotted using known concentrations of the reducing sugars to check for their corresponding responses.

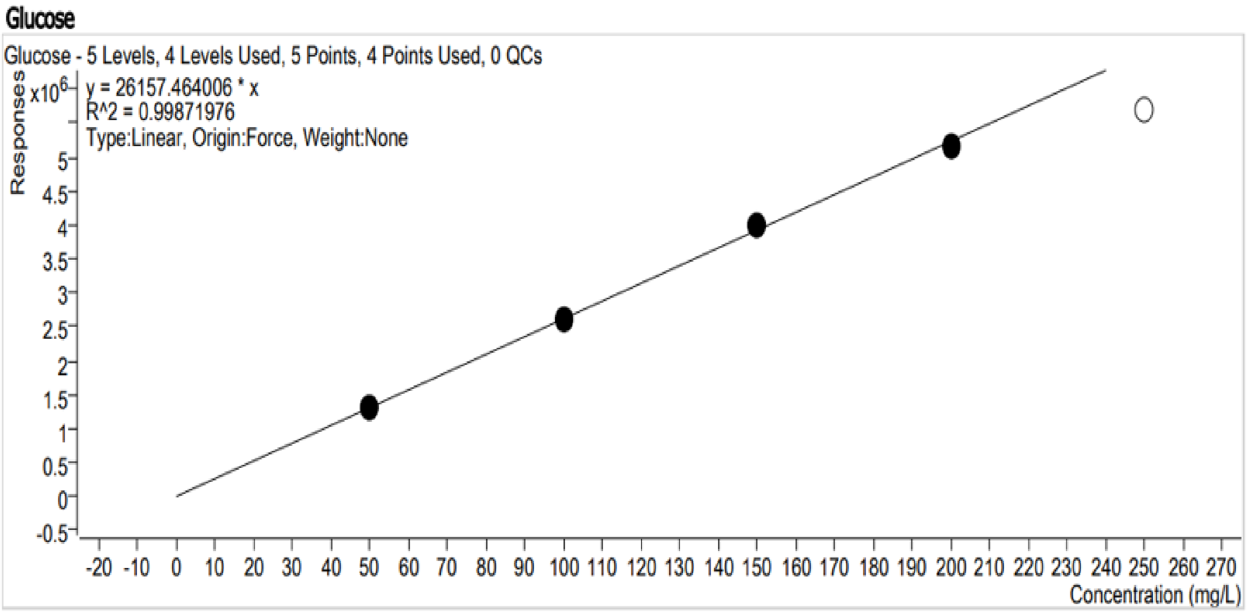

Figure 6: Standard curve plot from LCMS responses to 50, 100, 150, 200 and 250 mg/l concentration of glucose in mobile phase (80/20 acetonitrile/water with 0.1% ammonium).

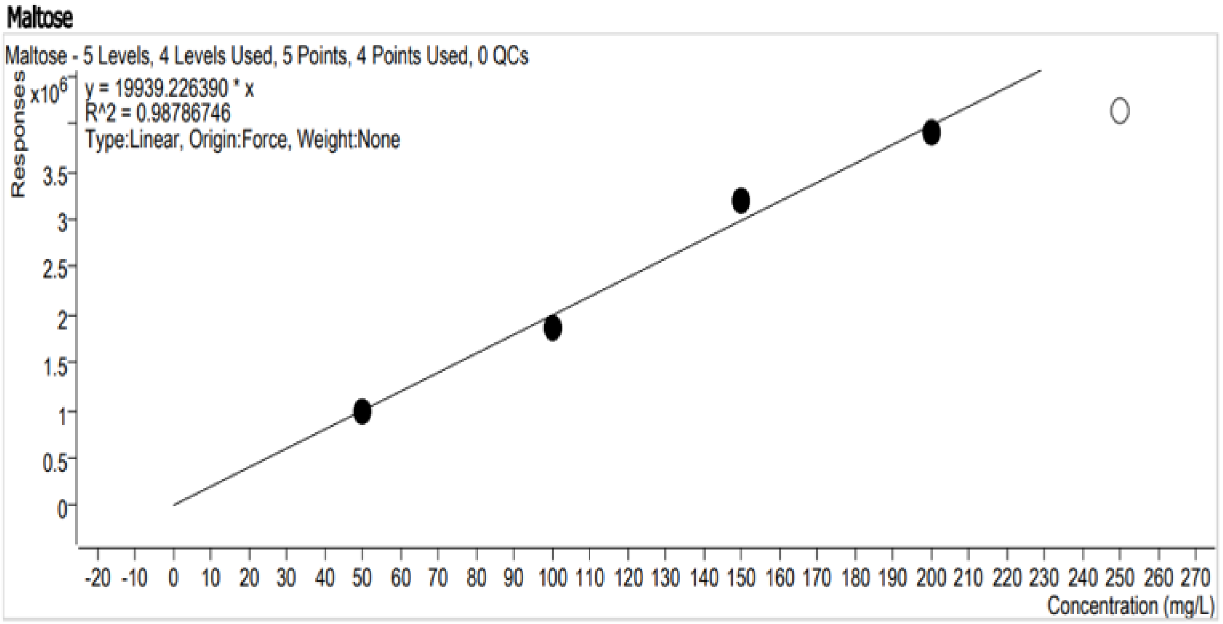

Figure 7: Standard curve plot from LCMS responses to 50, 100, 150, 200 and 250 mg/l concentration of maltose in mobile phase (80/20 acetonitrile/water with 0.1% ammonium).

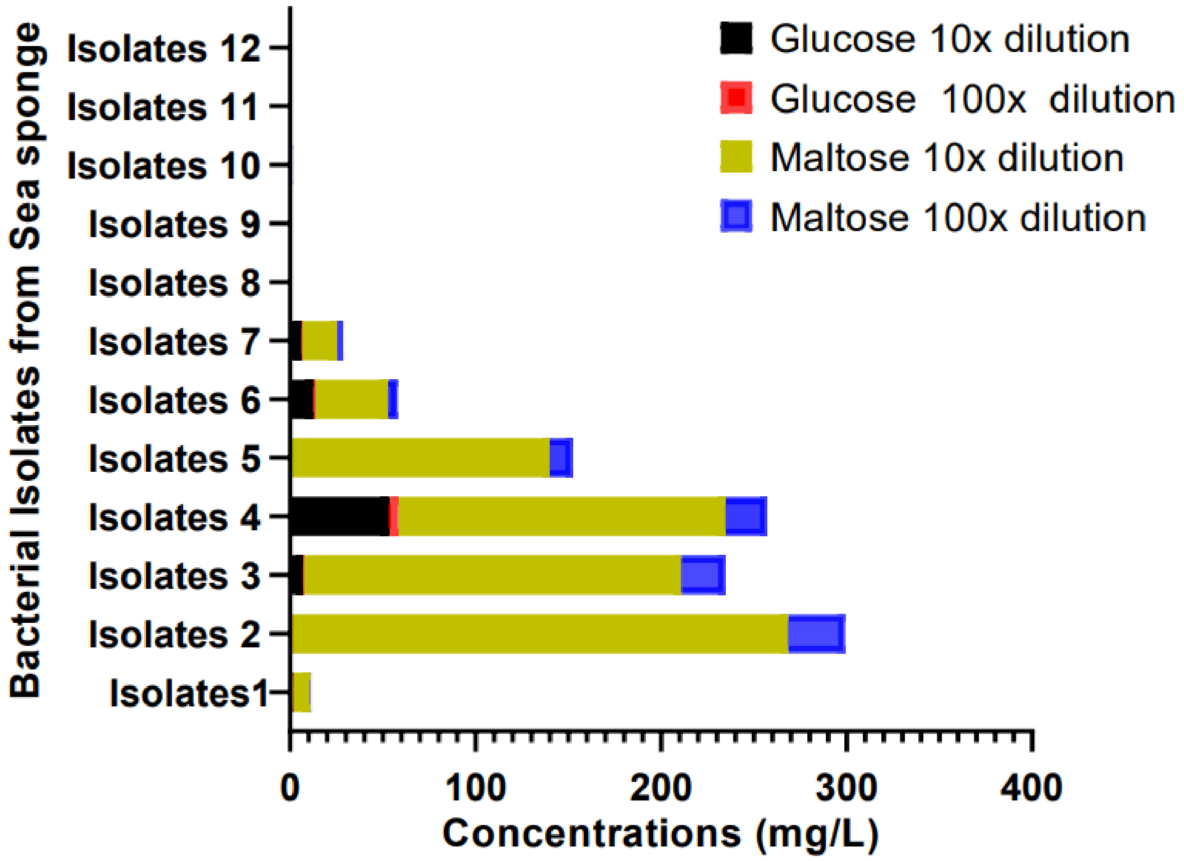

Figure 8: Glucose and maltose concentration (mg/l) from LC/MS analysis at x10 and x100 dilutions.

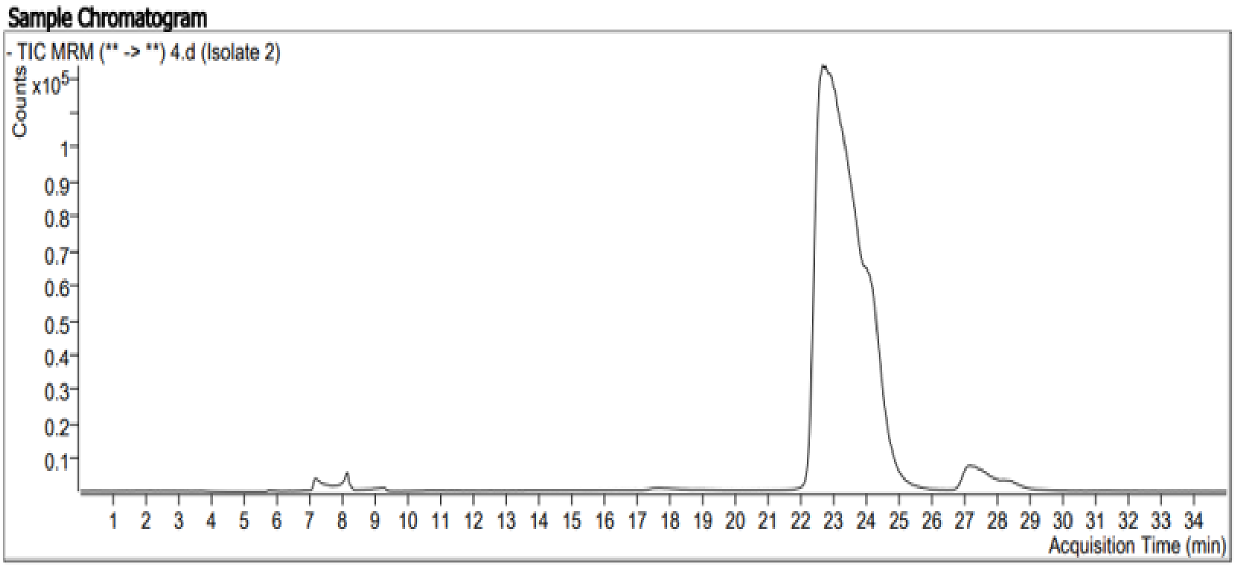

Compound	Transition	RT	Resp.	Final Conc	Units
Glucose	179.1 -> 59.0	17.680	29377	1.1231	mg/L
Maltose	341.1 -> 101.1	22.655	5328506	267.2374	mg/L

Figure 9: Chromatograph of maltose as major reducing sugar produced from starch hydrolysis by beta amylase from isolate 2.

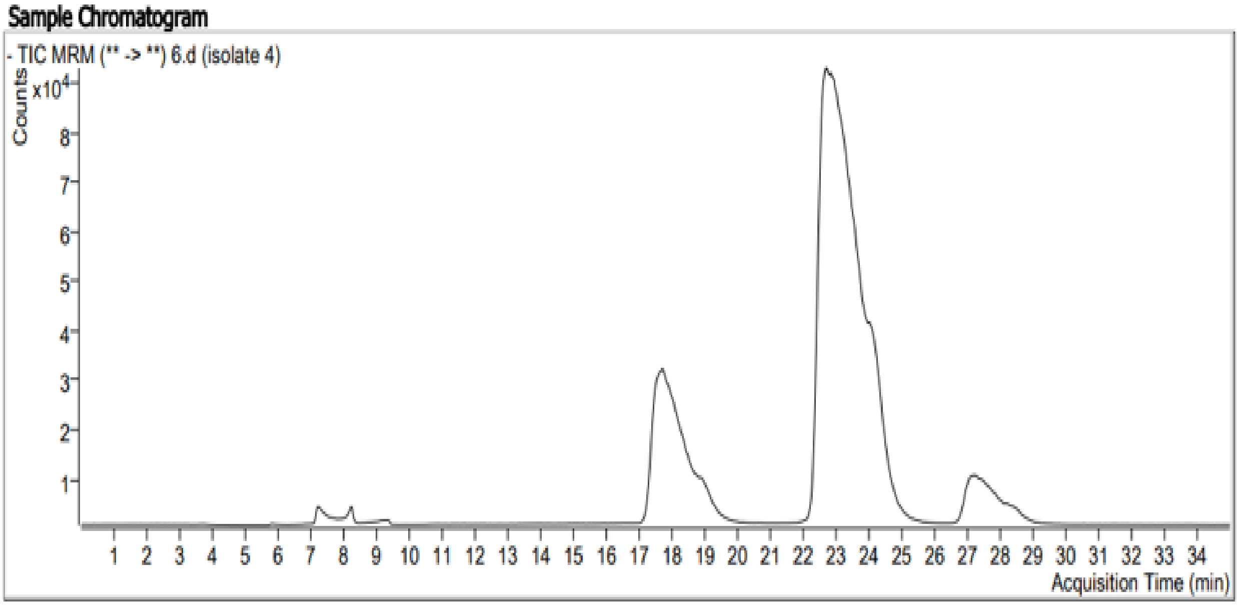

Compound	Transition	RT	Resp.	Final Conc	Units
Glucose	179.1 -> 59.0	17.709	1388555	53.0845	mg/L
Maltose	341.1 -> 101.1	22.698	3514066	176.2388	mg/L

Figure 10: Chromatographic view of Glucose and maltose; reducing sugar produced from starch hydrolysis by alpha amylase from isolate 4.

DISCUSSION

In this research, sponge-bacterial symbionts were cultured on starch agar medium (a medium used to differentiate amylase producer from non-amylase producers when flooded with iodine). From the growth of bacteria on this medium, and the extraction processes, it is observable that the marine sponge *H perlevis* served as host for microorganisms of various amylase producing capacities. Sequel to the isolation of 12 amylase positive bacteria, (figure 3) submerged liquid-state fermentation (SLF) technique was used for the extraction of amylase. This technique provided adequate environment for bacteria to feed, reproduce and release amylase to breakdown starch molecules into smaller reducing sugars.

The DNS analysis with the aid of a spectrophotometer at 540nm was used to quantify the level of starch hydrolysis in the medium. This method (DNS assay) was adopted since it is the most widely used methods for the quantification of reducing sugar [26]. After separating the bacterial cells from the crude enzyme and treating the crude enzyme with DNS, a reduction reaction is expected if starch has been hydrolysed (figure 5a). From the results (figure 5a and 5b) there was evidence that DNS (3,5 dinitrosalicyclic acid) had reacted with reducing sugar to produce 3-amino-5-nitrosalicyclic acid (ANS). The amount of ANS produced determined how deep or light the colouration of each crude enzyme treatment when compared to the control. The light amber-orange colour of DNS changes to a deep amber or red-brown colouration due to production of ANS. The level of reducing sugar produced by the hydrolysis of starch is expected to be directly proportional to the absorbance level (figure 5b).

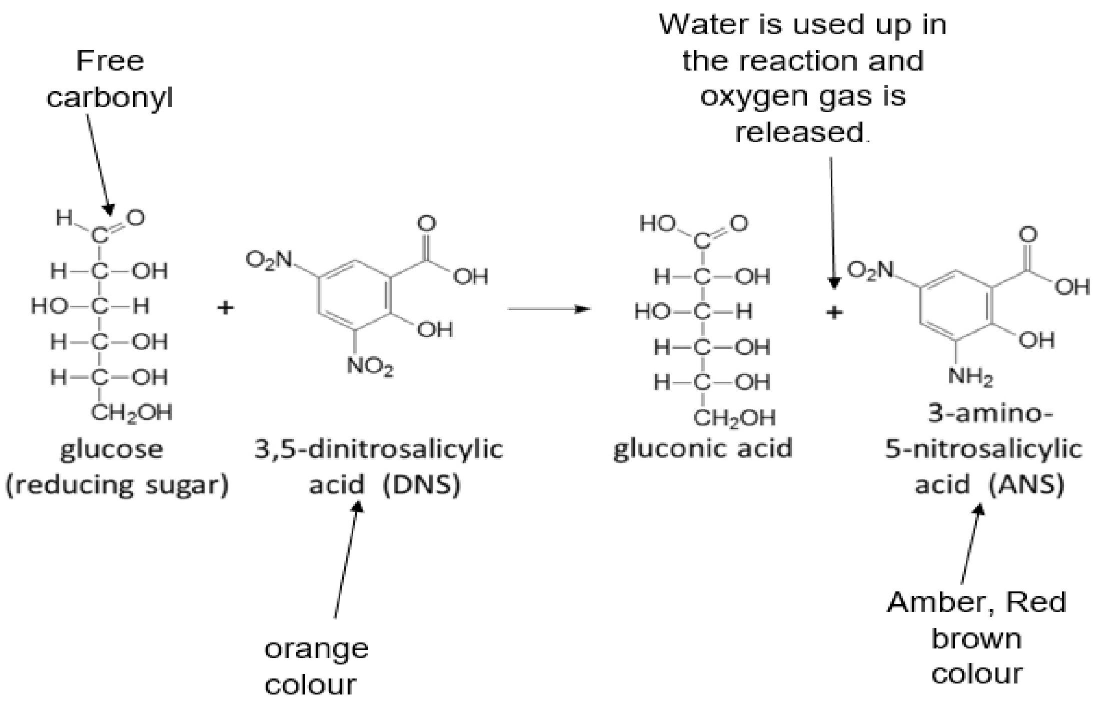

Figure 11 Molecular description of DNS reduction reaction with reducing sugar adopted from [27] with some modification.

At this point, the reducing sugars produced from the hydrolysis of starch by amylase were unknown. It was therefore necessary to conduct a more precise qualitative and quantitative analyses to know exactly which product (reducing sugar) were formed and at what concentrations using the liquid chromatography mass-spectrometry. From the LC-MS analysis of the crude enzyme (amylases) produced by the sponge bacterial symbionts, it was observed that out of the seven reducing sugars (glucose, maltose, fructose, galactose, mannose, lactose, and sucrose) which was used to optimise the machine, only glucose and maltose were identified as the major products of the starch hydrolysis. The result of the analyses (figure 8) at a x10^{-1} dilution, showed that isolates 2 (figure 9) had the highest concentration of maltose production at 267.237mg/l, followed by isolate 3 with 203.391mg/l, isolate 4 with 176.238 mg/l and isolate 5 with 139.938 mg/l. It was also observed that isolate 4 had the highest glucose concentration of 53.084 mg/l (figure 10) followed by isolate 6 with

12.309mg/l, isolate 3 with 6.906mg/l and isolate 7 with 5.909mg/l. From these results (figure 8 and 9) the formation of maltose as the major product of the hydrolysis of starch by amylase suggests that the amylase is a β-amylase (EC 3.2.1.2). This result agrees with the findings of [1-2] and implies that α-1,4-glucan linkages [28] in the starch were hydrolysed by the enzyme to from maltose. The formation of appreciable amount of both glucose and maltose as products of hydrolysis by amylase produced by isolate 3,4,6 and 7, suggest the amylase is an α-amylase (E.C.3.2.1.1). This result also agrees with the findings of [5] which signifies that alpha-1,4-glycosidic bonds have been hydrolysed by the enzyme to produce glucose and maltose as products.

Sponge bacterial symbionts form *H perlevis* is a good source for amylases production given the global distribution of the sponge around marine ecosystems [29]. In addition, marine enzymes are beginning to become preferred for biotechnological and other industrial application [30]. In the view of sustainability, the need for new sources of enzyme to replace inorganic sources cannot be over emphasised. The amylase produced by the marine sponge bacterial symbionts as biocatalyst for the hydrolysis of starch into glucose, maltose, and other starch moieties, encourages the practice of green and sustainable chemistry. Amylase producing sponge bacterial symbionts can be isolated within environmentally benign temperature range (10^0c to 37^0c). Both α-amylase (E.C.3.2.1.1) and β-amylase (EC 3.2.1.2) produced by the isolates functions with water which is an environmentally benign solvent [31].

Since *H perlevis* is widely distributed along coastal rockpools of marine water bodies, there are chances of exploiting the production of alpha and beta amylases from bacterial symbionts in *H perlevis* for commercial purposes. Sponge tissues serve as host for many microorganisms because of the symbiotic relationship that exist between the sponge and microorganisms like bacteria.

CONCLUSION

Various researchers have proposed enzyme mediated reaction as the key to green and sustainable chemistry [32]. Enzyme from marine microorganisms is a better way forward for a green and sustainable enzyme mediated production process.

The use of amylase from marine sponge *H perlevis* is encouraged as a sustainable practice given the global distribution of the marine sponge, the benefits associated with amylases from extreme environment like the marine environment, the economic potentials and ease of growth. This research supports the sustainable development goal of proper conservation and use of marine bodies and resources for a sustainable development. Achieving green and sustainable processes is not just guaranteed because an enzyme is involved [11]. There is need to monitor enzyme mediated processes, select environmentally benign options, and cost-effective processes etc to keep the process sustainable.

ACKNOWLEDGMENTS

We wish to appreciate the University of the West of England (UWE) Bristol United Kingdom for funding this research. We also appreciate the UWE Forensic LC/MS senior technician and the 2K6 Microbiology Technical team for their technical support.

REFERENCE:

1. Ray RR, Nanda G. Microbial β-amylases: biosynthesis, characteristics, and industrial applications. Critical reviews in microbiology. 1996 Jan 1;22(3):181-99.
2. Sundarram A, Murthy TP. α-amylase production and applications: a review. Journal of Applied & Environmental Microbiology. 2014;2(4):166-75.
3. Lal A, Cheeptham N. Starch agar protocol. American Society for Microbiology. 2012 Nov 1:1-9.

4. Dash BK, Rahman MM, Sarker PK. Molecular identification of a newly isolated Bacillus subtilis BI19 and optimization of production conditions for enhanced production of extracellular amylase. BioMed Research International. 2015 Jun 9;2015.
5. Montazer M, Harifi T. Introduction: textile finishing. Nanofinishing of Textile Materials; Woodhead Publishing: Cambridge, UK. 2018:1-7.
6. Kandra L. α-Amylases of medical and industrial importance. Journal of Molecular Structure: THEOCHEM. 2003 Dec 1;666:487-98.
7. Sivaramakrishnan S, Gangadharan D, Nampoothiri KM, Soccol CR, Pandey A. a-Amylases from microbial sources–an overview on recent developments. Food Technol Biotechnol. 2006 Mar 11;44(2):173-84.
8. Headon DR, Walsh G. The industrial production of enzymes. Biotechnology advances. 1994 Jan 1;12(4):635-46.
9. Alcalde M, Ferrer M, Plou FJ, Ballesteros A. Environmental biocatalysis: from remediation with enzymes to novel green processes. TRENDS in Biotechnology. 2006 Jun 1;24(6):281-7.
10. Anastas PT, Warner JC. Principles of green chemistry. Green chemistry: Theory and practice. 1998;29.
11. Paravidino M, Hanefeld U. Enzymatic acylation: Assessing the greenness of different acyl donors. Green Chemistry. 2011;13(10):2651-7.
12. Raveendran S, Parameswaran B, Ummalyma SB, Abraham A, Mathew AK, Madhavan A, Rebello S, Pandey A. Applications of microbial enzymes in food industry. Food technology and biotechnology. 2018 Mar;56(1):16.
13. Zhao Q, Li Y, Shen B, Zhao Q, Zhu L, Jiang L. UiO-66-Mediated Light-Driven Regeneration of Intracellular NADH in Clostridium tyrobutyricum to Strengthen Butyrate Production. ACS Sustainable Chemistry & Engineering. 2023 Feb 14;11(8):3405-15.
14. Baharum SN, Beng EK, Mokhtar MA. Marine microorganisms: potential application and challenges.
15. Zhang C, Kim SK. Research and application of marine microbial enzymes: status and prospects. Marine drugs. 2010 Jun 23;8(6):1920-34.

16. Otaiku AA. Bioremediation of Hydrocarbon Production Wastes using Environmental Biotechnology Inoculants (OBD-Plus®) Obagi, Niger Delta, Nigeria: Findings. (MSc). Enugu State university of science and technology Nigeria. 2007 Aug.
17. Taylor MW, Radax R, Steger D, Wagner M. Sponge-associated microorganisms: evolution, ecology, and biotechnological potential. Microbiology and molecular biology reviews. 2007 Jun;71(2):295-347.
18. Webster NS, Taylor MW. Marine sponges and their microbial symbionts: love and other relationships. Environmental microbiology. 2012 Feb;14(2):335-46.
19. Bell JJ, Bennett HM, Rovellini A, Webster NS. Sponges to be winners under near-future climate scenarios. Bioscience. 2018 Dec 1;68(12):955-68.
20. Sipkema D, Franssen MC, Osinga R, Tramper J, Wijffels RH. Marine sponges as pharmacy. Marine biotechnology. 2005 Jun;7:142-62.
21. Bell JJ. The functional roles of marine sponges. Estuarine, coastal and shelf science. 2008 Sep 10;79(3):341-53.
22. Ramírez GA, Bar-Shalom R, Furlan A, Romeo R, Gavagnin M, Calabrese G, Garber AI, Steindler L. Bacterial aerobic methane cycling by the marine sponge-associated microbiome. Microbiome. 2023 Dec;11(1):1-5.
23. Manconi R, Pronzato R. How to survive and persist in temporary freshwater? Adaptive traits of sponges (Porifera: Spongillida): A review. Hydrobiologia. 2016 Nov;782:11-22.
24. Kennedy J, Baker P, Piper C, Cotter PD, Walsh M, Mooij MJ, Bourke MB, Rea MC, O'Connor PM, Ross RP, Hill C. Isolation and analysis of bacteria with antimicrobial activities from the marine sponge Haliclona simulans collected from Irish waters. Marine biotechnology. 2009 Jun;11:384-96.
25. Fishman LD. *The effect of commercial starch blockers on starch hydrolysis in vitro* (Master's thesis, California State University, Northridge).
26. Deshavath NN, Mukherjee G, Goud VV, Veeranki VD, Sastri CV. Pitfalls in the 3, 5-dinitrosalicylic acid (DNS) assay for the reducing sugars: Interference of furfural and 5-hydroxymethylfurfural. International journal of biological macromolecules. 2020 Aug 1;156:180-5.

27. Applegate KB, Cheek PR, Inlow JK. Analysis of kombucha to teach biochemical concepts and techniques to undergraduate students. Biochemistry and Molecular Biology Education. 2019 Jul;47(4):459-67.

28. Hii SL, Tan JS, Ling TC, Ariff AB. Pullulanase: role in starch hydrolysis and potential industrial applications. Enzyme research. 2012;2012.

29. Turner TL. The marine sponge Hymeniacidon perlevis is a globally distributed invasive species. bioRxiv. 2020 Feb 13:2020-02.

30. Parte S, Sirisha VL, D'Souza JS. Biotechnological applications of marine enzymes from algae, bacteria, fungi, and sponges. InAdvances in food and nutrition research 2017 Jan 1 (Vol. 80, pp. 75-106). Academic Press.

31. Ni Y, Holtmann D, Hollmann F. How green is biocatalysis? To calculate is to know. ChemCatChem. 2014 Apr;6(4):930-43.

32. Itoh T. Enzymatic Reactions using Ionic Liquids for Green Sustainable Chemical Process; Stabilization and Activation of Lipases. The Chemical Record. 2023 Jan 11:e202200275.
