# Supplementary material for: Amylase production from marine sponge Hymeniacidon perlevis; potentials sustainability benefits
Source: PLoS One. 2023 Dec 21;18(12):e0294931. doi: 10.1371/journal.pone.0294931 (PMC10734944; doi:10.1371/journal.pone.0294931)
Supplement: Attachment — Submitted filename: Response to reviewers comments.docx [file pone.0294931.s002.docx]

29^th^ July 2023

**Re: PONE-D-23-14066**

Dear Dr Mohamed,

We thank the referees for their detailed and helpful comments regarding our manuscript entitled: “Amylase Production from Marine Sponge *Hymeniacidon Perlevis*; Potentials Sustainability Benefits” and for the editorial comments. We note that all the reviewers recognise that the paper has merit and recommend publication subject to revisions. We have now fully addressed all of the editorial comments and the comments of all reviewers fully as outlined in detail below and look forward to the publication of our manuscript in PLOS ONE.

Editorial Comments

We thank the editor for their useful comments and have made all the changes suggested and have also consulted the editorial checklist for guidance on format and syntax. As requested, we have removed all redundant files from our submissions and have only included current and relevant versions. We have uploaded a ‘response to reviewers’ letter’ containing a point-by-point response to the points raised by the Editor and/ or Reviewers. We have replaced Figure 1 with our own picture of a sponge fragment as requested and therefore will not need copyright permission. Also, we have also included the statement on the funder in the cover letter as requested. We have now included in the method session additional information regarding the permits we obtained for the work and the full name of the authority that approved the field site access. We have removed all funding-related statements from the manuscript as requested. There are no amendments to the original funding statement.

**Reviewer 1:** We thank referee one for their positive comments and for their useful critiques which we have now addressed in full as outlined below:

***Point 1:*** The manuscript has been proofread.

***Point 2:*** We thank the referee for this comment**.** The subject of this paper is amylase production from starch hydrolysis by symbiotic bacterial isolates from the marine sponge (*H. perlevis*) and its sustainability significance. Based on the kind of bonds hydrolysed and the sugars formed from the starch hydrolysis we were able to characterise the type of amylase produced into alpha and beta amylases using LCMS. To the best of our knowledge, no one has researched on the amylase produced by this sponge species, this is novel and a major contribution to this field of study given the cosmopolitan nature of this sponge species.

***Point 3:*** Lines 185-192 to the end of the discussion contain information on amylase characterisation**:** Characterization of amylase has been added to the methods. Amylases were characterised as Alpha and beta amylases based on Colorimetric assay and the sugars were identified by LC-MS. This information is in the results and discussion section. See point 17 below for more information.

***Point 4:*** Citations have been updated

***Point 5:*** Line 102-105**:** The main aim of the study has been updated in the last part of the introduction.

***Point 6****:* Line 29: Bracket has been added to “3.5µm, 4.6x150mm” in line 29

***Point 7:*** The sentence in lines 28-30 has been restructured as requested

***Point 8:*** Lines 49-68**:** The entire sentence has been rewritten**.** The main reason for this paragraph, however, is because starch is the major substrate used in this study and serves as an indicator for the presence or absence of amylase in the samples analysed. Hence, we believe it is useful to include a statement that makes the connection between Starch and Amylase to provide some context for readers in the introduction.

***Point 9:*** Lines 56-57: Reference added

***Point 10:*** Lines 59-61: the phrase “Alpha-amylase has the enzyme commission number “E.C.3.2.1.1”. has been removed.

***Point 11:*** Lines 97-105: a short paragraph about the current study has been added

***Point 12:*** Lines 115-117: Figure 2 has been replaced with a picture of the collected sponge and is referred to in the text.

***Point 13:*** Line 121: Information on how the sponge tissue was blended has been updated.

***Point 14:*** Lines 137-145: The paragraph has been rewritten.

***Point 15****:*  The manuscript has been proofread

***Point 16****:* Lines 343-356: Clarification on the novelty of our findings. The subject of this paper is amylase production from starch hydrolysis by symbiotic bacterial isolates from the marine sponge *H.perlevis*, and its sustainability significance. Analysis of the hydrolysed bonds and the resultant sugars formed using LCMS provided matching fingerprint evidence that aligned with those of alpha and beta amylases. Hence, we are confident that this study successfully isolated starch hydrolysing bacteria from our sea sponge species with significant amylase activities. Fig 4 shows a positive amylase test with a zone of clearance to indicate starch hydrolysis after flooding the plate with iodine. This further confirmed amylase activity in the isolates. To the best of our knowledge, this study is the first to establish amylase activities from isolates of this sponge species. We believe this is novel.

***Point 17:*** Lines 187-192 to the end of the discussion contain information on amylase characterisation**:** Characterization of amylase has been added to the methods. Alpha and beta amylases were found to be the enzymes produced. This information is in the results and discussion section. The result section contains information on the quantitative characterisation of amylase activities using DNS colourimetric assay. The isolates as can be seen in Figs 5a and b showed significant amylase enzyme activity. This was further confirmed by LCMS and the maximum activity of isolates was achieved in 4% starch and salt concentration.

***Point 18:*** Lines 151-163: Section re-written and more details about Colourimetric assay have been added as requested

***Point 19:*** The term “product” has been replaced with crude enzyme or simple sugar throughout the manuscript.

***Point 20:*** Lines 167-177: Information on Liquid Chromatography Mass Spectrometry procedure/methodology has been updated.

***Point 21:*** Lines 194- 198: Information on the statistical software used as well as the number of replicates has been updated

***Point 22*:** Lines 201-212: The term “pure culture” has been replaced with “pure colonies”. This session has also been rewritten.

***Point 23:*** Line 137 and Line 167: Submerged liquid fermentation is referred to in the methods session.

***Point 23:*** Figure 11 moved from the discussion to the methods section as requested.

***Point 24:*** Lines 349- 362: The conclusion has been updated with a clearer summary of the work and results obtained.

Yours Sincerely,

Rachael U Chidugu-Ogborigbo PhD

Praise Nnaji MSc

Ruth H Morse PhD

Emmanuel Adukwu PhD
